# Digital technology in maize nutrient management research in northern Nigeria amid COVID-19 pandemic

**DOI:** 10.1038/s41598-024-58740-1

**Published:** 2024-04-06

**Authors:** Kamaluddin T. Aliyu, Bello M. Shehu, Adam M. Adam

**Affiliations:** 1 International Maize and Wheat Improvement Center (CIMMYT), LA Boulevard Rd, Lusaka, Zambia; 2 Department of Soil Science, Bayero University Kano (BUK), Kano, 70001 Nigeria; 3 Centre for Dryland Agriculture (CDA), Bayero University Kano, Kano, 70001 Nigeria

**Keywords:** COVID-19, Digital tools, Maize, Site-specific recommendation, Nutrient expert, Ecology, Environmental sciences, Mathematics and computing

## Abstract

The COVID-19 pandemic has been a life threatening and spreads wildly with physical human contact. Physical distancing is recommended by health experts to prevent the spread; thus, agronomic research has to be designed in conformity to this preventive standard during the pandemic. Consequently, this study was designed to evaluate the reliability of using digital tools in nutrient management research amid the COVID-19 pandemic in northern Nigeria. Fifty extension agents (EAs) were selected across 15 LGAs of Kaduna and Kano states. The EAs were trained on how to generate fertilizer recommendation using an android mobile phone-based nutrient expert (NE), to measure farmers’ field sizes using UTM Area measure mobile phone app, and open data kit to record, submit and aggregate data during the exercise. Each EA covered 50 farms, where two nutrient management practices—one determined by the farmers: farmer fertilizer practice (FFP), and the other generated using the NE were evaluated. Results show that around 90% of the farmers have an average field size of 1.13 ha. All selected farmers used improved maize varieties for planting, among which 21% been able to use the exact recommended or lower seed rate. Use of inorganic fertilizer was 33% higher than the average recommended NE rate, while average yield of the NE fields was 48% higher than for the FFP. The results of this study indicate that yield can be improved with site-specific nutrient management (SSNM) extension approach. The SSNM using digital tools as the NE seem promising and befits to agronomic research in northern Nigeria amid the COVID-19 pandemic.

## Introduction

Maize has been a very important food security crop worldwide and in many sub-Saharan African (SSA) countries including Nigeria^1,2^. In Nigeria, maize production is largely concentrated in the northern Nigeria savanna zones (Guinea and Sudan) which accounts for about 80% of the maize total production in the country^3^. Although the savannas have a potential maize yield of over 5 t ha^−1^, however, average yields measured in farmers’ fields is hardly up to 2 t ha^−1^; as a result of some production constraints^4^.

Nutrient management has been the most critical constraint affecting maize production in the savanna zones^5^. Despite the weak extension system in the country^2^, considerable effort is frequently directed to maize nutrient management advisory^6^. However, the nutrient management advisory is regional based “blanket” and in that sense it ignores the significant dynamism in fertilizer application requirement based on field- and season-specific conditions and their temporal variabilities^7,8^.

Optimal nutrient management strategy is considered a workable option to improve maize productivity in the northern Nigeria savannas^9^. Several studies^10,11^ among others suggested that adoption of site-specific nutrient management enhances maize yield by increasing nutrient use efficiency and nutrient response. However, the low extension agent (EA) to farmer ratio remains a major obstacle in the effective dissemination of the site-specific nutrient management advisory delivery in northern Nigeria. In addition to that, the emergence of COVID-19 in Nigeria resulted to a strict mandatory 14-day initial total lock-down in early 2020 as a measure to curb the spread of the disease^12^. Then at that time the lock-down was extended to May; which represents a critical farming period. Since the measures to stop the spread of the COVID-19 are of social distancing which aimed to reduce physical human contacts, hence, agronomic research activities have to be designed to conform to the COVID-19 preventive measures—the “*new normal*”.

The global revolution of digital technology has transformed many aspects of life; and is envisaged to as well transform agricultural research and advisory services to real-time data tracking. As of 2019, 67% of the global population had subscribed to mobile devices, of which 65% were smartphones—with the fastest growth in sub-Saharan Africa^13^. In 2019, 204 billion apps were downloaded^14^, and as of January 2020, 3.8 billion people actively used social media^15^. Leveraging on those technological breakthroughs, in this study a nutrient management research using digital android-based tool known as the nutrient expert (NE) was piloted in Kano and Kaduna states of northern Nigeria to provide a resilient platform for conducting nutrient research amid the COVID-19 crisis.

The NE is a user-friendly interactive, scalable nutrient management extension tool developed by the ‘Taking Maize Agronomy to Scale (TAMASA)’ project in Nigeria, Tanzania and Ethiopia^2^. The NE generates site-specific nutrient management recommendation by estimating nutrient yield response following the principles of the quantitative evaluation of the fertility of tropical soils (QUEFTS) model and site agronomic information^10,11^. The tool recommends NPK fertilizer application based on 4R’s of nutrient use principles which promotes application of fertilizer using right source (i.e., the type of fertilizer needed), at the right rate (i.e., the amount of fertilizer), right time (i.e., the timing of fertilizer application), and in the right place (i.e., most proper application method)^10^. The input data required to generate the recommendations include the actual farmer field size, farmer’s current crop management practices (inorganic and organic fertilizer use, previous crop and yield, crop residue management, etc.), characteristics of the growing environment (risk of flood/drought), soil fertility (using indicators such as pH, soil texture, colour, depth, organic matter, problems of salinity etc.). This information is utilized by the tool to estimate the risks associated with reaching the attainable yield for a farmer’s location^10^. Farmer may however opt for a yield lower than the attainable yield as the target yield based on his/her financial situation and production preference by allowing recommendations to be adjusted according to their available budget. After synthesizing the input data, the NE produces a printable output of N, P and K recommendation to guide for achieving a target maize yield, and a simple profit analysis based on prevailing market cost for seed and fertilizer to compare farmers’ current practice and the NE generated recommendations. In light of the COVID-19 pandemic, this approach of nutrient management was tested to underline the relevance of digital extension tools as a strategy to provide a resilient platform for remotely conducting agronomic research.

## Materials and methods

### The study area

**Figure 1:**
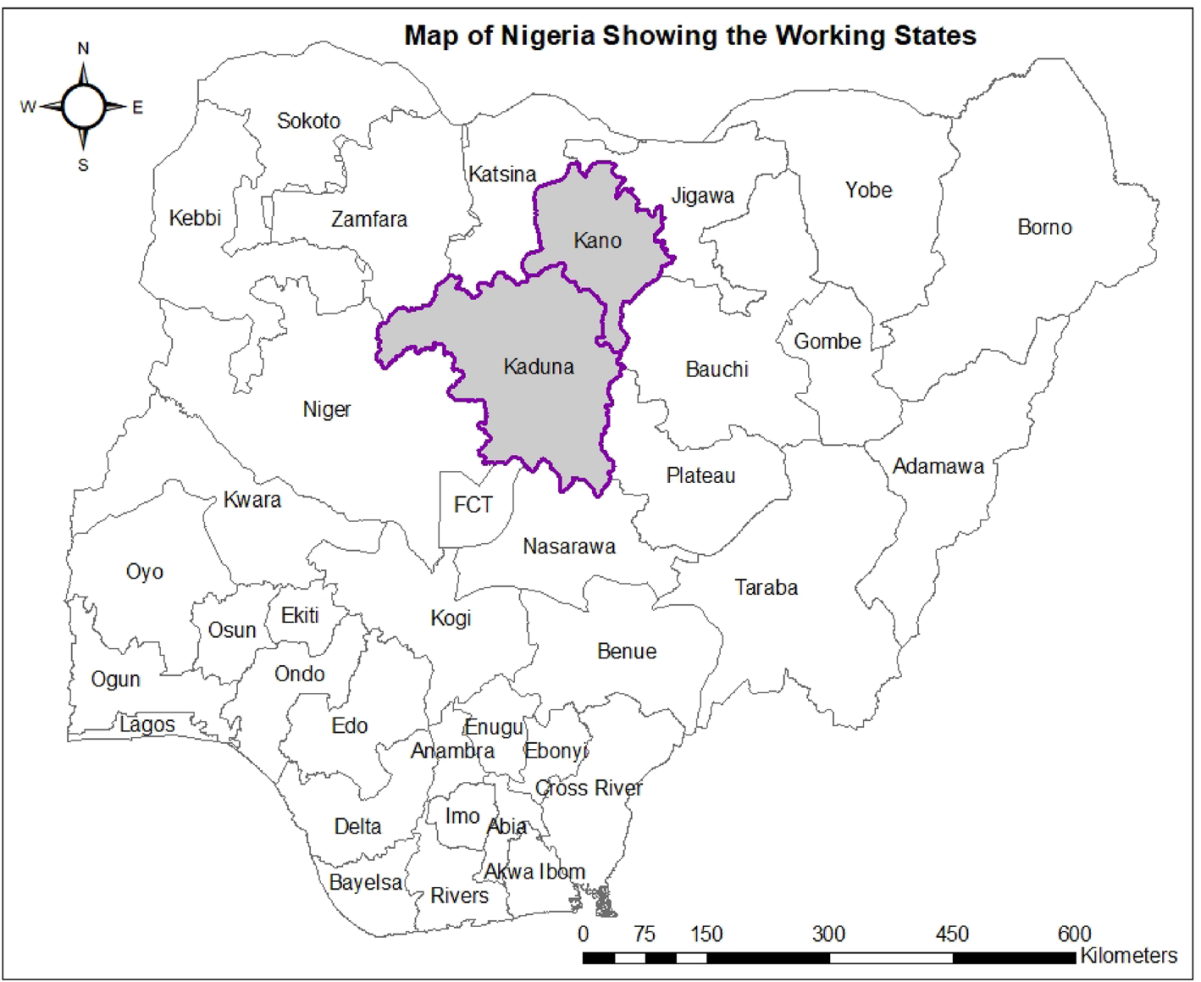
Map of Nigeria showing the study location/States for the implementation of site-specific nutrient management extension advisory research. Map generated using ArcGIS Desktop: Release 10.3.1 Redlands, CA: Environmental Systems Research Institute. www.esri.com/en-us.

The study was conducted in 2020 cropping season in Kaduna and Kano States located within Guinea and Sudan Savanna agro-ecologies in northern Nigeria. These two States (Fig. 1) were purposively selected because of their relative importance in maize production, and to reduce travelling distance during the study implementation stages. Fifteen local government authorities (LGAs)—2 in Kano and 13 in Kaduna were selected based on availability of contact and institutional linkage to extension agents (EAs).

Maize is the most important cereal crop in the selected LGAs. It is cropped predominantly in mixture and under rotation with legumes like soybean or cowpea, and with other cereal like rice and sorghum^16^. Traditional tillage system using hoe and or animal traction is very commonly done to conserve soil moisture and pulverize the soil. Fertilizer application rate for maize is averagely low at 50 N kg ha^−1^ due to several financial and social factors^2^, while retention of crop residues on fields is not very common because residues are often used as livestock feed, for fencing and fuel^17,18^.

### Implementation of the nutrient recommendation advisory

The implementation of the study was done through the extension agents platform of Sasakawa Africa Association (SAA) office in Nigeria. SAA is an agriculture-based extension organization with head office in Tokyo. The SAA Nigeria office played the initial role of pre-selecting extension agents (EAs) in Kano and Kaduna States based on the criteria of been able to operate android smart phones. At least three EAs were selected from each LGA of Kaduna and one each from the two Kano state LGAs to give a total of 50 EAs across the states. Each EA was provided with an android mobile phone and trained on three android-based application software. First, the EAs were trained to generate and interpret nutrient recommendation using the NE. Emphasis was geared toward proper understanding on how to reasonable guide farmers to provide close to real estimates of the main input data required (listed under introduction section above) to generate the recommendation. Second, because most farmers do not have a precise measurement of their farm size (farm area size is a key requirement in NE), the EAs were also trained to use UTM Area measure app to measure farmers’ field size. The UTM Area Measure is a free accessible smart phone-based app which uses satellite and GPS to measure distance of a directory or area/perimeter by walking round the boundary. Measurements using the app can be saved, imported and exported in various formats, and can be used in GIS software environment. Thirdly, because of the restricted movement sanctioned by the states to curtail the spread of COVID-19, the EAs were trained on using another open-source mobile app known as the Open Data Kit (ODK) for routine data collection. The ODK is a tool for designing digital forms for any form of data collection. Forms in the ODK can be designed to collect alpha-numerical, photos, GPS location, audio-visual information and etcetera^19^. Once downloaded, ODK forms works offline. Forms can be saved and edited before they are sent to a central cloud data host created by the user. If GPS coordinate is customized in the forms, easy mapping of individual data point can be viewed on the central server dashboard. Summary of submitted forms (total number of forms submitted, time and date of submissions) are provided on real-time basis, while alpha-numerical data are shown in a tabular or graphical format. Data can also be downloaded in various formats such as xlx, csv, tiff, etc. The use of ODK in this study was to provide effective monitoring of data collection while movement was restricted. A WhatsApp forum was also created to facilitate quick question and answer session among the EAs, and between the scientists leading the study.

### The NE fertilizer recommendations

During the training, the EAs were instructed to target at most 50 farmers that will receive the NE fertilizer recommendation in the current season. Selection of farmers was left to the decision of the EAs based on their prior knowledge of farmers’ readiness for adopting innovative technology and compliance with EAs farming advisory. To evaluate the performances of the NE against the farmer farm practice (FFP), the farms were divided into two sections, with size of each section measured. Farmers were allowed to practice their conventional practices of nutrient management but using same maize variety as on the NE section. The NE recommended practice was implemented on the other section of the farm under the supervision of the EA. One-week time line was agreed upon for generating the target number of recommendations in order to reduce variability in yield that may be caused by delayed planting. Total of 1,197 NE fertilizer recommendations were generated and monitored through 46 (out 50) trained EAs. Field monitoring and evaluation tours were conducted in the last weeks of August 2020 and October, respectively by the implementation team to interact with farmers.

### Routine data collection

Data were collected on the date of planting, seed type and variety used for planting, date, type and amount of fertilizer applied. Incidence of disease outbreak was also considered where necessary. When such happens the EAs sent pictures of the affected fields through the WhatsApp forum to get real-time suggestions from the scientists in the team. Other data on timing of farm activities like weeding (number and type) and harvesting were also recorded. At harvest, the cobs were cut and allowed to dry for two weeks before they were shelled. Grain yield was estimated from each fertilizer practice by weighing the grains, and using the measured field area the yield was converted to per hectare.

### Data presentation

Data collected were curated using descriptive statistical tools, and outliers for each type of date were removed by plotting the data in box plot with whiskers. Values outside the length of the whiskers were removed. The data are presented as histogram, bar chart, and pie chart depending on the intended purpose. All plotting was done in Microsoft excel 2016.

### Ethical declaration

This study did not involve the use of human nor animal subjects.

## Results and discussion

**Figure 2:**
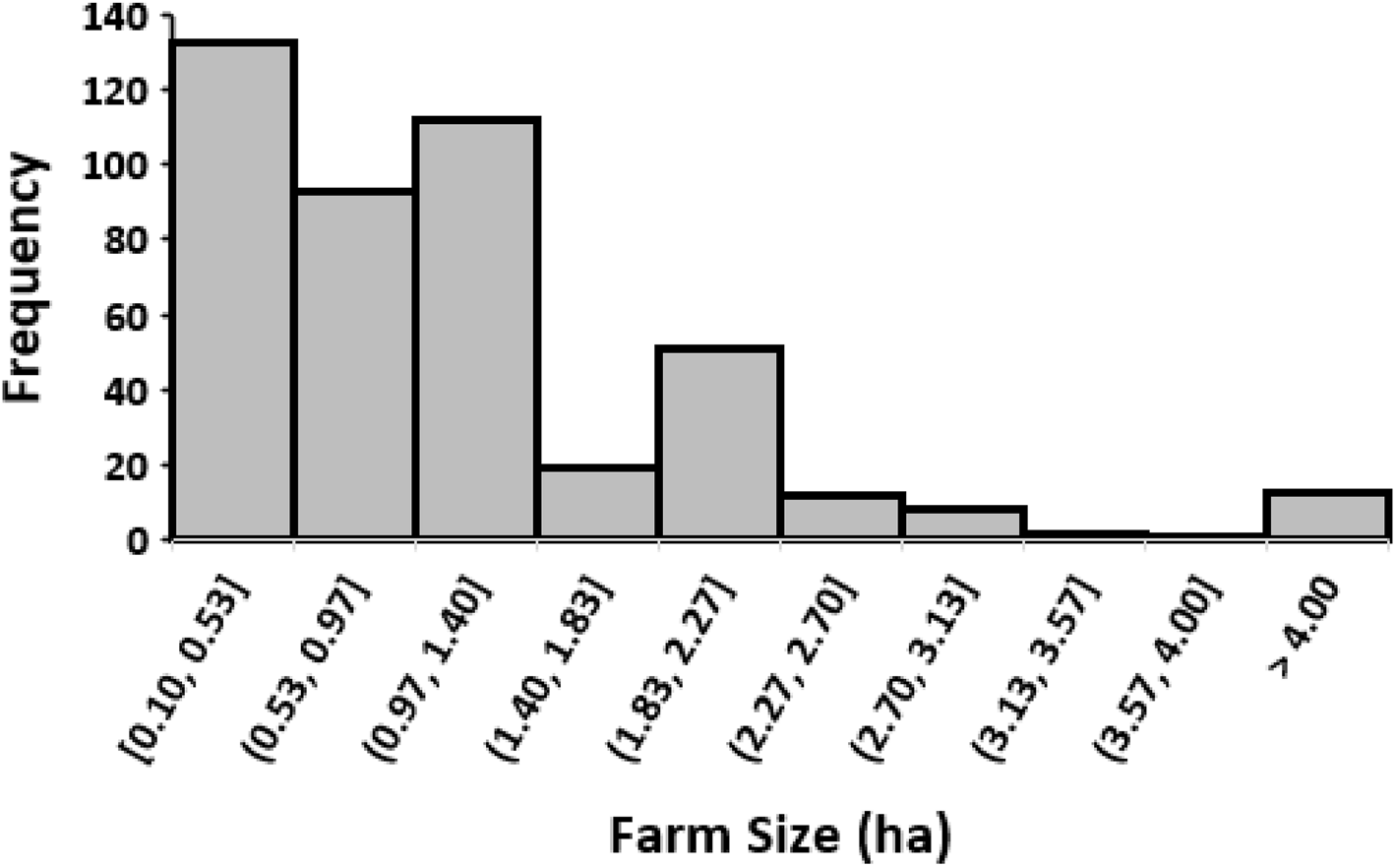
Histogram showing the distribution of farmers’ field sizes in the study area.

Result of the measured farmers’ field sizes (Fig. 2) show that the smallest farms among the selected farmers was 0.1 ha, while 10 ha was the largest; and occurred only once. More than 90% of the farm sizes were ≤ 2.0 ha; which is the typically reported size owned by smallholder farmers in the region. Among these farmers about 29% have lower than 0.5 ha. Similarly, small farm size holdings in this area have been reported^2,11,20^. Land in this area is reported to be acquired through inheritance in over 86% of the cases reported by Don^21^. Through this, the lands are sub-divided into the segment for all heirs. This system is believed to constitute the reason for the highly fragmented farms in the area.

**Figure 3:**
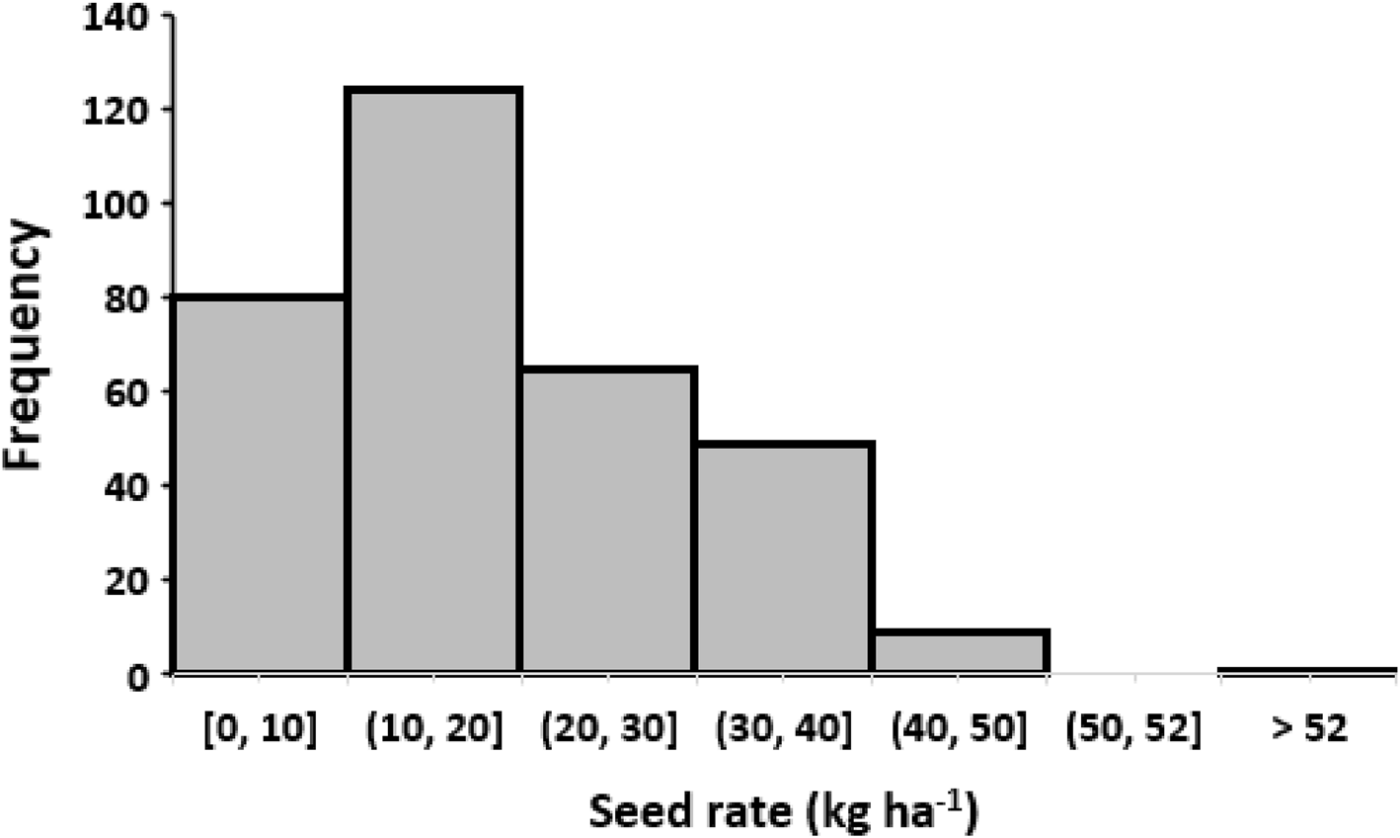
Histogram showing the distribution of actual maize seed rate (kg ha^−1^) used by the farmers under Farmers Fertilizer Practice (FFP) compared to the NE (Nutreint Expert) recomended rate of 20–25 kg ha^−1^.

**Figure 4:**
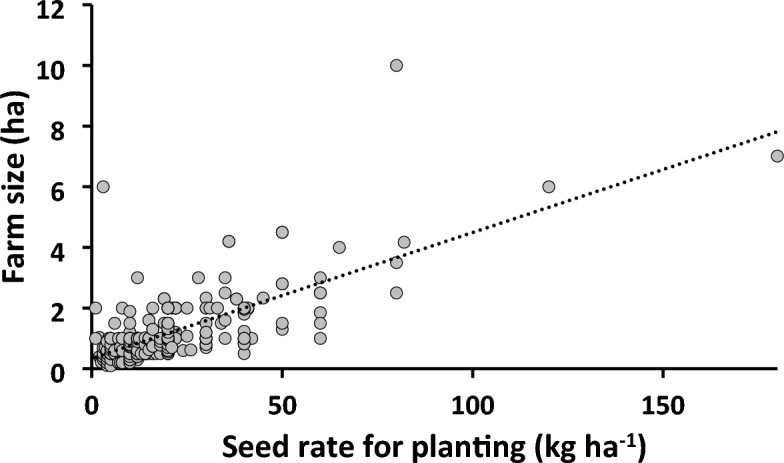
A linear relationship between farm size(ha) and seed rate used (kg ha^−1^) by farmers under FFP.

The recommended rate of maize seed to plant a hectare was 25 kg. This rate was also considered the standard practice and was adopted in the NE recommendation. However, for the farmers practice (FFP), farmers determined their practice based on their convention. Data on seed rate used for planting show a wide variation among the FFP fields (Fig. 3). Although a reasonable portion of the farmers (20.1%) used the NE recommended seed rate, 13.3% used higher than the recommended rate. But, in majority of the FFP cases (66.1%), farmers used lower seed rate, with 10–19 kg ha^−1^ being the most consistent of the cases. In all the cases, type of seed used was (improved) either hybrid or OPV. Wider and un-even spacing seem to be the reason for the low seed rate among farmers which indicates sub-optimal plant population. Finding of Carlsson et al.^22^ confirmed that optimum plant population has greater yield potential than unevenly spaced stands. Several researchers have reported that close planting is a key management technique that increases maize yields and reduces to a large extent the yield variability commonly observed between farmers’ fields^23^. A relationship between farm size and seed rate per hectare of the FFP (Fig. 4) indicates a positive correlation with a coefficient of determination (R^2^) of 0.5. The figure shows that farmers with ≤ 2.0 ha farm holdings used lower seed rates in 85% of the cases, while double the recommended rate was used for farms that are ≥ 4 ha. As stated earlier, majority of the farmers in this study were smallholder, and they are typically characterized by low input farming^24^. This category of farmers is normally described by limited access to income, small asset ownership, limited access to services and credit institution, and farmer associations, which constrain their farm budget^2^. This is in line with a large part of the technology adoption literature pointing to less-endowed farmers being more likely to reject improved farm technologies due constraint farm budget^25,26^.

**Figure 5:**
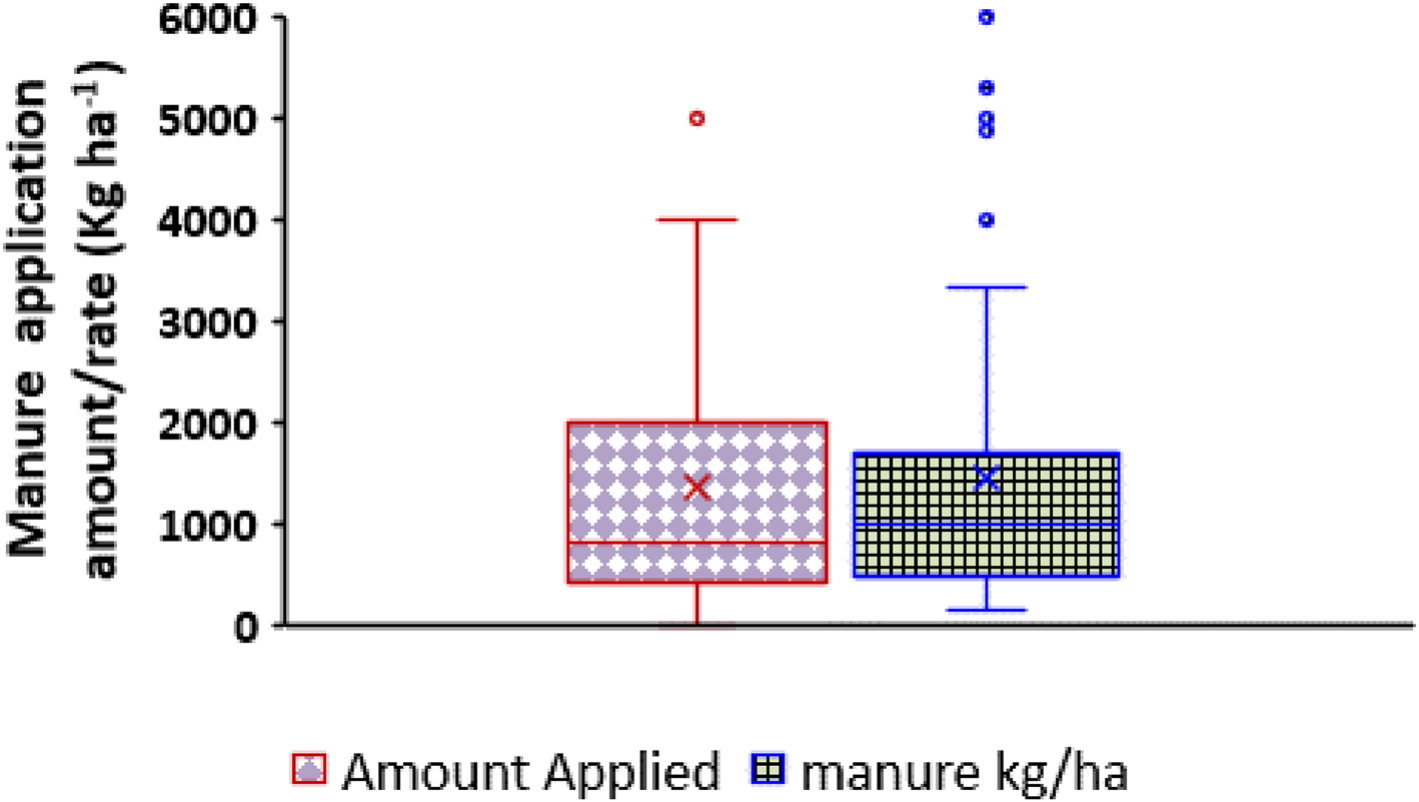
A box plot showing the distribution of amount of manure used (kg) and manure application rate (kg ha^−1^) by the farmers in the study.

**Figure 6:**
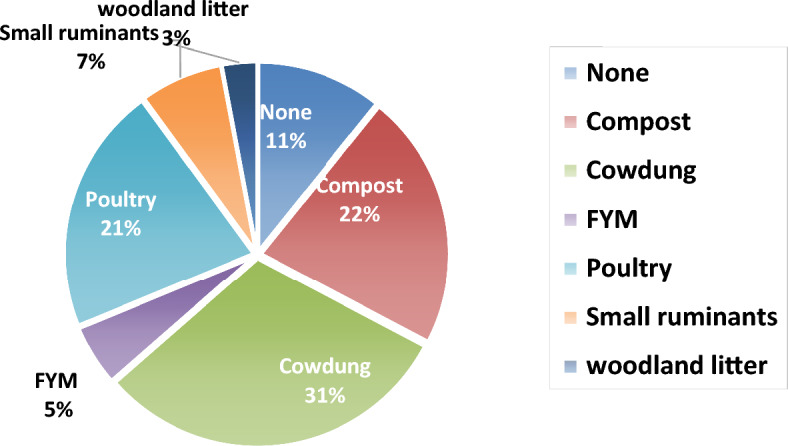
Pie chart showing the composition of various organic manure types used by the farmers in the study.

Irrespective of farm size, use of organic manure in the study area was on the average below 1000 kg; with a median value of 1200 kg (Fig. 5). Low use of organic manure among farmers has been reported by Aliyu et al.^16^ in the same area, it was also reported to diminish attainable maize yield^27,28^. Cow dung manure was the most predominantly used (in 31% of cases) organic manure among the farmers (Fig. 6) probably due to common access to cattle kept for farm works^16,17^. Compost and poultry manure types were used in 22 and 21% of the scenarios. Manures from small ruminants and farm yard respectively constituted 7 and 5% of manure type used, while 11% of the farmers did not use any form of organic manure. However, for large farms (≥ 4 ha), the average manure application rate was 1200 kg ha^−1^, with a median of 1800 kg ha^−1^, while few farmers applied up to 6000 kg ha^−1^ (Fig. 5).

**Figure 7:**
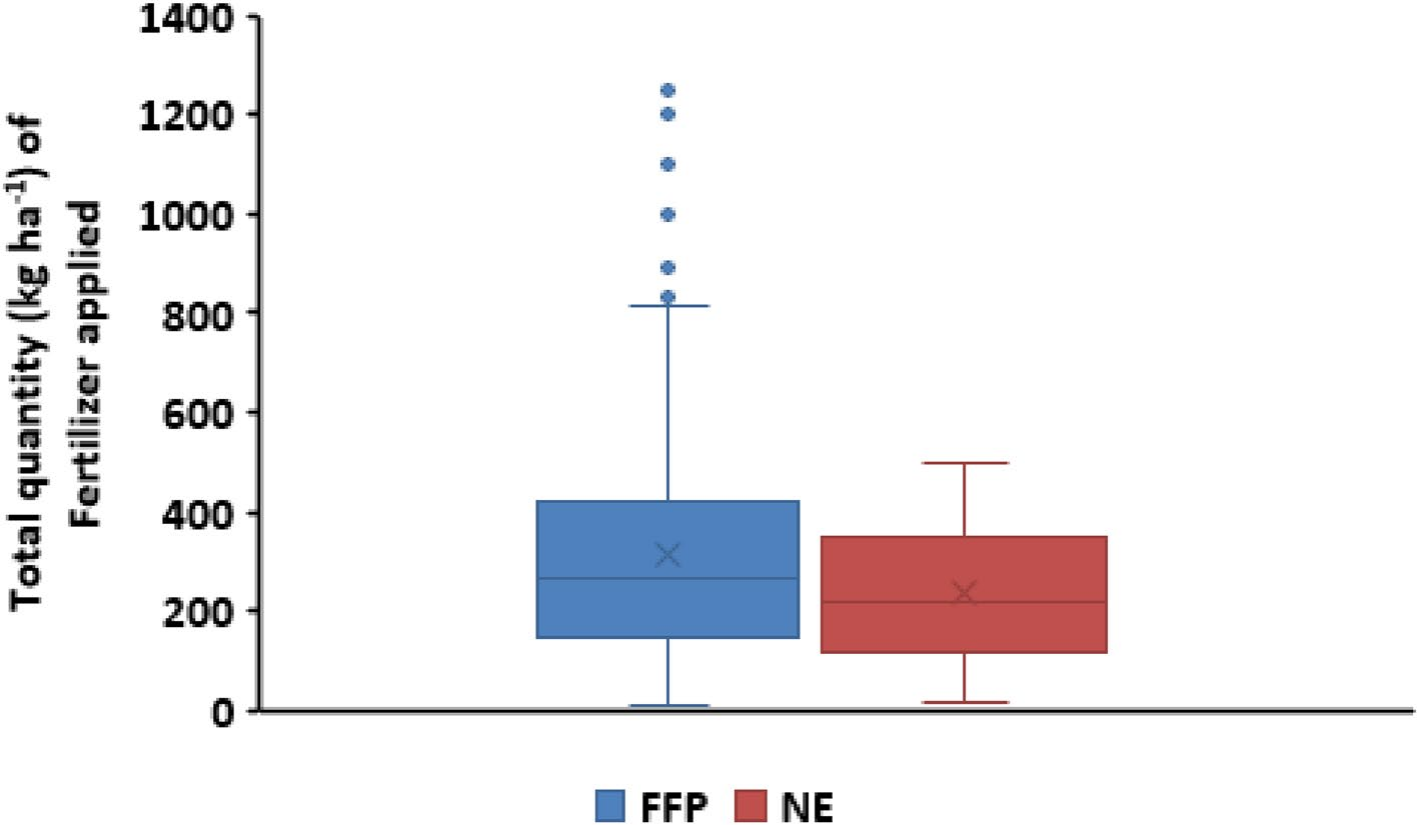
Box plot comparing total amount (kg ha^−1^) of fertilizer (NPK and Urea) used under the FFP and NE nutrient managements.

Use of inorganic fertilizer (NPK and urea combined) for the FFP varied widely among the farmers (Fig. 7). The variable use of fertilizer is considered to be the result high cost due to the banning of fertilizer products importation by the government and closure of fertilizer blending plants due to COVID-19. Other documented factors are low access to credit, high transportation costs, behavioural constraints such as risk and time preferences, poor knowledge of fertilizer use, as well as to a variable poor yield response to fertilizer applications^6,7,29^. Average fertilizer applied using the FFP was around 300 kg ha^−1^, with about 50% of farmers applying up to 380 kg ha^−1^. This rate was 33% higher than the average recommended NE rate (210 kg ha^−1^) for most farms. In a similar vein, Liverpool–Tasie et al.^20^ reported a range between 15 and 25% higher fertilizer application around Kano and Kaduna States.

**Figure 8:**
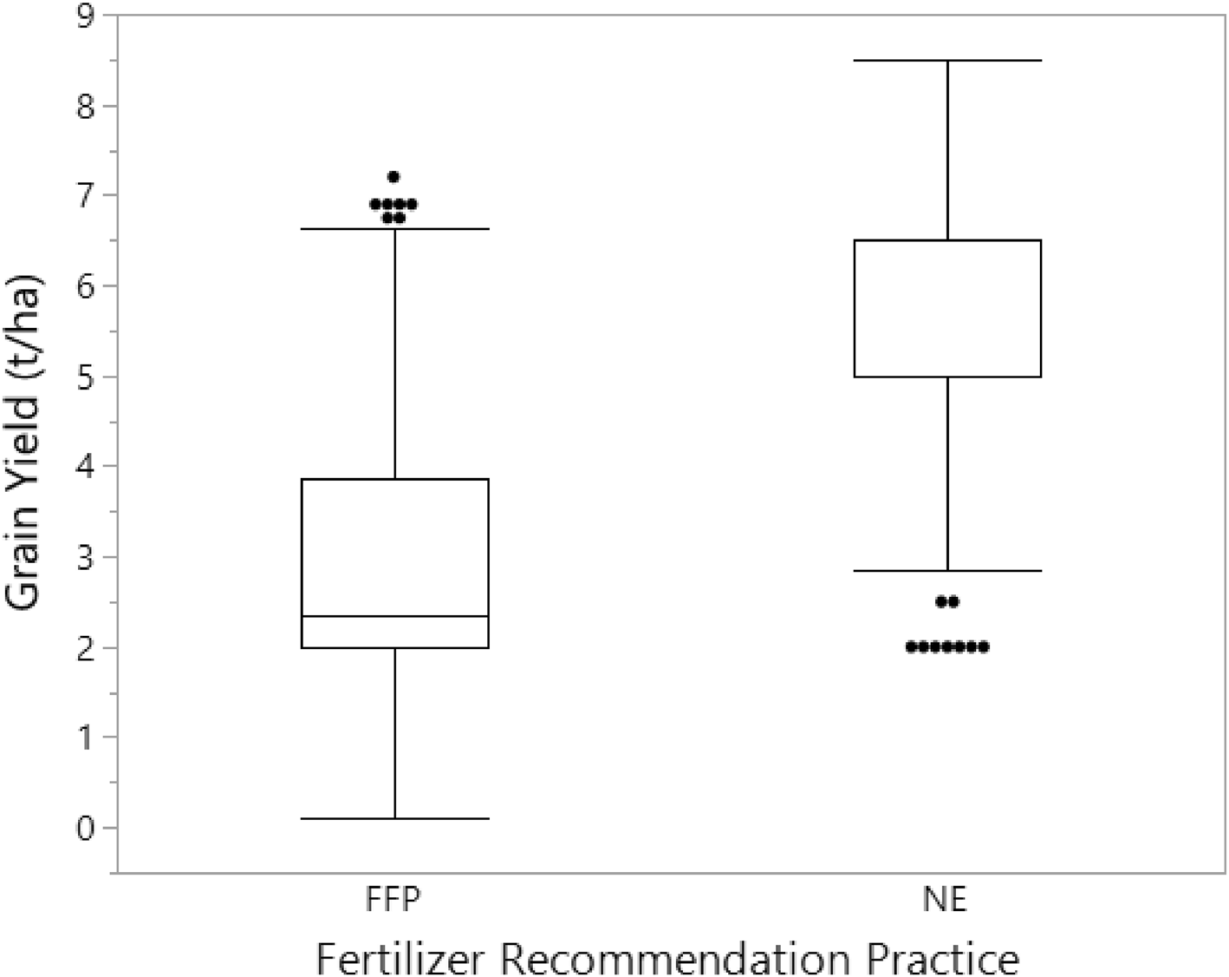
Distribution of maize grain yield under the FFP and the NE recommended practice.

Maize grain yield seems to likewise varied more under the FFP than the NE; though this also show some variation among the farms. Average yield of the FFP was 2900 kg ha^−1^; and this is 48% lower compared to that of the NE (5500 kg ha^−1^) (Fig. 8). The average maize yield of the FFP is higher than the reported national average yield of 1500–2000 kg ha^−1^^30^. The yield from the NE recommendation is however consistent with most reported yields from well manged research fields^8,11,31^. This indicates that the NE tool recommendation can be relied upon for nutrient management extension and for improved yield.

## Conclusions

This study was designed to assess the reliability of using digital nutrient management and extension tools in conducting agronomic research amid the COVID-19 in northern Nigeria savannas. The study discovered that majority of the extension workers can conduct agronomic nutrient management research with minimal supervision, but a rigorous training is needed to achieve that. More accurate estimate of farmers’ field sizes enabled the implementation of the site-specific nutrient management (SSNM) recommendation. More than 90% of the farmers were smallholders with average field size of 1.13 ha. All the selected farmers planted improved maize varieties; either hybrid or OPV, with 85% among them using the exact recommended or lower seed rates. Use of organic manure was low as expected, while that of inorganic was 33% higher than the average recommended NE rate. While average yield of the NE fields was 48% higher than for the FFP, the yield was also more inconsistent among the FFP plots. The preliminary data analysis presented in this article indicates that higher yield can be achieved with SSNM approach. The SSNM using digital tools as the NE seems promising and befit the realization process for e-extension revolution in northern Nigeria. However, one of the main difficulties in deploying digital nutrient management and extension technologies during the COVID-19 in the northern Nigerian savannas, according to the extension agents, is travelling to farmers’ fields. We believed that more research should thoroughly assess these difficulties in order to maximise the study’s relevance and influence in related agricultural systems as well as anywhere else.

## Data Availability

Data set used in this study will be made available upon reasonable request from B.M. Shehu.

## References

[1] van Ittersum MK (2016). Can sub-Saharan Africa feed itself?. PNAS.

[2] Oyinbo O (2019). Farmers’ preferences for high-input agriculture supported by site-specific extension services: Evidence from a choice experiment in Nigeria. Agric. Sys..

[3] Fakorede, M. A. B. & Akinyemiyu, O. A. Climatic change: effects on maize production in a tropical rainforest location. In: *Maize Revolution in West and Central Africa* (eds*.* Badu-Apraku, B., Fakorede, M. A. B., Ouedraogo, M., Carsky, R. J. & Menkir, A*.*) 272 – 282 (Proceedings of regional maize workshop. Cotonou 2003).

[4] Abdoulaye T, Wossen T, Awotide B (2018). Impacts of improved maize varieties in Nigeria: Ex-post assessment of productivity and welfare outcomes. Food Sec..

[5] Jibrin MJ, Kamara AY, Ekeleme F (2012). Simulating planting date and cultivar effect on dryland maize production using CERES maize model. Afr. J. Agric. Res..

[6] Ande OT (2017). Status of integrated soil fertility management (ISFM) in southwestern Nigeria. Int. J. Agric. Res..

[7] Tarfa BD, Wortmann CS, Sones K (2017). Optimizing fertilizer use within the context of integrated soil fertility management in Nigeria. Fertilizer use optimization in sub-Saharan Africa.

[8] Shehu B, Merckx R, Jibrin MJ, Kamara AY, Rurinda J (2018). Quantifying variability in maize yield response to nutrient applications in the northern Nigerian savanna. Agronomy.

[9] Garba II, Jibrin JM, Kamara AY, Adnan AA, Bassam AL (2020). Response of maize to secondary nutrients and micronutrients in the Guinea savanna of Nigeria. J. Agronomy.

[10] Pampolino MF, Witt C, Pasuquin JM, Johnston A, Fisher MJ (2012). Development approach and evaluation of the nutrient expert software for nutrient management in cereal crops. Comp. & Electron. in Agric.

[11] Rurinda J (2020). Science-based decision support for formulating crop fertilizer recommendations in sub-Saharan Africa. Agric. Syst..

[12] Ahmed I, Isa AB, Abdullahi Y, Poom K, Wiyada K (2020). Analysis of Caputo fractional-order model for COVID-19 with lockdown. Adv. Diff. Equ..

[13] GSMA. The mobile economy https://www.gsma.com/mobileeconomy/ (2020).

[14] Perez, S. App stores saw record 204 billion app downloads in 2019, consumer spend of $120 billion. TechCrunch https://techcrunch.com/2020/01/15/app-stores-saw-record-204-billion-app-downloads-in-2019-consumer-spend-of-120-billion/ (2020).

[15] Kemp, S. D. Global digital overview. https://datareportal.com/reports/digital-2020-global-digital-overview (2020).

[16] Aliyu KT (2020). Delineation of soil fertility management zones for site-specific nutrient management in the Maize belt region of Nigeria. Sustainability.

[17] Manyong VM, Makinde KO, Sanginga N, Vanlauwe B, Diels J (2001). Fertilizer use and definition of farmer domains for impact-oriented research in the northern Guinea savanna of Nigeria. Nutr. Cycl. Agroecosyst..

[18] Akinola AA, Ayedun B, Abubakar M, Sheu M, Abdoulaye T (2015). Crop residue usage and its determinants in Kano State. Nigeria. J. Dev. Agric. Econ..

[19] Tonnang HEZ (2020). Rapid acquisition, management, and analysis of spatial maize (*Zea mays* l.) phenological data—Towards ‘big data’ for agronomy transformation in Africa. Agronomy.

[20] Liverpool-Tasie LSO, Omonona BT, Sanou A, Ogunleye WO (2017). Is increasing inorganic fertilizer use for maize production in SSA a profitable proposition? Evidence from Nigeria. Food Policy.

[21] Don NI (1984). The system of land rights in Nigerian agriculture. Ame. J. Econ. & Sociol..

[22] Carlsson F, Köhlin G, Mekonnen A, Yesuf M (2005). Are Agricultural Extension Packages What Ethiopian Farmers Want?: A stated preference analysis.

[23] Echarte L (2000). Response of maize kernel number to plant density in Argentinean hybrids released between 1965 and 1993. Field Crop. Res..

[24] Anderson JM, Ingram JSI (1993). Tropical soil biology and fertility (TSBF): A hand book of methods.

[25] Kuehne G (2017). Predicting farmer uptake of new agricultural practices: a tool for research, extension and policy. Agric. Syst..

[26] Lambrecht I, Vanlauwe B, Merckx R, Maertens M (2014). Understanding the process of agricultural technology adoption: Mineral fertilizer in Eastern DR Congo. World Dev..

[27] Zingore S, Murwira HK, Delve RJ, Giller KE (2007). Influence of nutrient management strategies on variability of soil fertility, crop yields and nutrient balances on smallholder farms in Zimbabwe. Agric Ecosyst. Environ..

[28] Kwari JD, Kamara AY, Ekeleme F, Omoigui L, Bationo A, Waswa B, Okeyo JM, Maina F, Kihara JM (2011). Soil fertility variability in relation to the yields of maize and soybean under intensifying cropping systems in the tropical savannas of northeastern Nigeria. Innovations as key to the green revolution in Africa: exploring the scientific facts.

[29] Sanni SA, Doppler W (2007). Socio-economic determinants of household fertilizer use intensity for maize-based production systems in the northern Guinea savannah of Nigeria. J. Applied Sci..

[30] Food and Agriculture Organization of the United Nations (FAOSTAT). Available online: faostat3.fao.org/download/Q/QC/E (accessed on 1 February 2020).

[31] Kihara J (2016). Understanding variability in crop response to fertilizer and amendments in sub-Saharan Africa. Agric. Ecosyst. Environ..

